# Onchocerciasis Transmission in Ghana: Persistence under Different Control Strategies and the Role of the Simuliid Vectors

**DOI:** 10.1371/journal.pntd.0003688

**Published:** 2015-04-21

**Authors:** Poppy H. L. Lamberton, Robert A. Cheke, Peter Winskill, Iñaki Tirados, Martin Walker, Mike Y. Osei-Atweneboana, Nana-Kwadwo Biritwum, Anthony Tetteh-Kumah, Daniel A. Boakye, Michael D. Wilson, Rory J. Post, María-Gloria Basañez

**Affiliations:** 1 London Centre for Neglected Tropical Disease Research, Department of Infectious Disease Epidemiology, Imperial College London, London, United Kingdom; 2 Natural Resources Institute, University of Greenwich at Medway, Chatham Maritime, Kent, United Kingdom; 3 MRC Centre for Outbreak Investigation and Modelling, Department of Infectious Disease Epidemiology, Imperial College London, London, United Kingdom; 4 Department of Vector Biology, Liverpool School of Tropical Medicine, Liverpool, United Kingdom; 5 Council for Scientific and Industrial Research, Water Research Institute, Accra, Ghana; 6 Ghana Health Service, Accra, Ghana; 7 Noguchi Memorial Institute for Medical Research, University of Ghana, Legon, Ghana; 8 School of Natural Sciences and Psychology, Liverpool John Moores University, Liverpool, United Kingdom; 9 Disease Control Department, London School of Hygiene and Tropical Medicine, London, United Kingdom; Creighton University, UNITED STATES

## Abstract

**Background:**

The World Health Organization (WHO) aims at eliminating onchocerciasis by 2020 in selected African countries. Current control focuses on community-directed treatment with ivermectin (CDTI). In Ghana, persistent transmission has been reported despite long-term control. We present spatial and temporal patterns of onchocerciasis transmission in relation to ivermectin treatment history.

**Methodology/Principal Findings:**

Host-seeking and ovipositing blackflies were collected from seven villages in four regions of Ghana with 3–24 years of CDTI at the time of sampling. A total of 16,443 flies was analysed for infection; 5,812 (35.3%) were dissected for parity (26.9% parous). Heads and thoraces of 12,196 flies were dissected for *Onchocerca* spp. and DNA from 11,122 abdomens was amplified using *Onchocerca* primers. A total of 463 larvae (0.03 larvae/fly) from 97 (0.6%) infected and 62 (0.4%) infective flies was recorded; 258 abdomens (2.3%) were positive for *Onchocerca* DNA. Infections (all were *O*. *volvulus*) were more likely to be detected in ovipositing flies. Transmission occurred, mostly in the wet season, at Gyankobaa and Bosomase, with transmission potentials of, respectively, 86 and 422 L3/person/month after 3 and 6 years of CDTI. The numbers of L3/1,000 parous flies at these villages were over 100 times the WHO threshold of one L3/1,000 for transmission control. Vector species influenced transmission parameters. At Asubende, the number of L3/1,000 ovipositing flies (1.4, 95% CI = 0–4) also just exceeded the threshold despite extensive vector control and 24 years of ivermectin distribution, but there were no infective larvae in host-seeking flies.

**Conclusions/Significance:**

Despite repeated ivermectin treatment, evidence of *O*. *volvulus* transmission was documented in all seven villages and above the WHO threshold in two. Vector species influences transmission through biting and parous rates and vector competence, and should be included in transmission models. Oviposition traps could augment vector collector methods for monitoring and surveillance.

## Author Summary

The World Health Organization (WHO) aims at eliminating onchocerciasis by 2020 in selected African countries. The success of elimination using ivermectin treatment alone will depend on several interacting factors including baseline endemicity, treatment coverage and vector species mix. In Ghana, transmission persists despite prolonged control. We investigated entomological determinants of this persistence. Blackflies were collected from seven villages with 3–24 years of ivermectin treatment. A total of 12,196 flies was dissected, with 463 larvae (all *Onchocerca volvulus*) in 97 infected and 62 infective flies. Transmission indices in the wet season, at Gyankobaa and Bosomase, amounted to, respectively, 86 and 422 infective larvae/person/month after 3 and 6 years of ivermectin treatment. Infection levels at these villages were over 100 times the WHO threshold of one L3/1,000 parous flies. At Asubende, an infective fly was caught among ovipositing flies in nearby breeding sites, indicating that infection was just over the WHO threshold despite extensive ivermectin and vector control. Spatial and seasonal vector species composition influences the magnitude of transmission indices through variations in biting and parous rates, and vectorial competence and capacity, and should be reflected in transmission models. Oviposition traps could enhance vector collection for transmission monitoring and surveillance.

## Introduction

The London Declaration on Neglected Tropical Diseases (NTDs) [1] and the World Health Organization’s (WHO) road map to accelerate progress for overcoming the impact of NTDs [2] have set goals for the elimination of human onchocerciasis by 2020 in selected African countries. Based on the results of epidemiological studies conducted in some foci of Mali, Senegal and Nigeria [3,4,5], it has been suggested that 14–17 years of annual (or biannual) ivermectin treatment may lead to local elimination of the infection reservoir in the absence of vector control. The repeatability of these achievements depends, in part, on the initial level of onchocerciasis endemicity, geographical and therapeutic coverage, treatment compliance and frequency, parasite susceptibility to ivermectin, and the intensity and seasonality of transmission, including the species composition of the simuliid vectors [6].

Previous reports assessing the feasibility of onchocerciasis elimination have concluded that although ivermectin mass drug administration (MDA) alone would help to eliminate the public health burden of onchocerciasis, it would not lead to elimination of infection in most foci, with the possible exception of areas of low endemicity [7]. However, more recent and encouraging results in areas of moderate to higher endemicity [3,4,5], have spurred the African Programme for Onchocerciasis Control (APOC) to shift its goals from morbidity control to local elimination of *Onchocerca volvulus* where possible [8]. Recognising the need to understand the nature and extent of transmission zones, APOC and WHO have emphasized the importance of conducting entomological studies on the determinants and feasibility of elimination [8,9,10]. Current WHO guidelines state that parasite levels within the vector need to be below a threshold of one L3 larva per 1,000 parous flies [11]. However, understanding how this measurement relates to the rate of transmission assessed via the biting rate, the infectious biting rate, the parous rate and the transmission potential, and importantly, how it varies with vector species composition and season, is vital for accurate monitoring and interpretation of this threshold [8].

Ghana was originally a country under the umbrella of the Onchocerciasis Control Programme in West Africa (OCP), which operated between 1974 and 2002, and was initially a vector control programme [12,13]. Vector control activities started in 1975 in the onchocerciasis savannah foci of northern and central Ghana, but the southern forest foci were not part of the programme [9]. When the microfilaricidal drug ivermectin was licensed for human use in 1987 [14,15], Ghana was one of the first countries to commence MDA. In particular, community trials were conducted in the then highly hyperendemic focus of Asubende (initial microfilarial prevalence of 80%) [16], where vector control had taken place but was suspended during the ivermectin distribution pilot study in the late 1980s. When the OCP ceased operations in 2002, the persistence of onchocerciasis at Asubende required this focus to be part of the so-called Special Intervention Zones, which maintained extensive coverage with ivermectin leading to dramatic reductions in infection intensity and prevalence [17].

In 2007, Osei-Atweneboana and co-workers [18] reported on the epidemiological situation in Ghana after the closure of the OCP and observed that despite vector control, and 19 years of annual ivermectin treatment, some communities exhibited high microfilarial prevalence and intensity (measured as the community microfilarial load) [19]. This was subsequently attributed to adult female worms being less responsive to the anti-fecundity effects of multiple treatments with ivermectin in some communities [20], but others pointed out the possibility of programmatic causes such as poor coverage permitting significant residual transmission [21,22,23]. Concerned by these findings, the NTD Programme of the Ghana Health Service initiated biannual ivermectin distribution in some communities in 2009 [6,24]. From 2003, ivermectin distribution was also extended to include endemic areas in Ghana which had not previously been included in the OCP.

Motivated by the need to understand the feasibility of elimination in Ghana, and in particular the entomological determinants of transmission persistence despite prolonged control, we conducted a study on the transmission of onchocerciasis in areas both within and outside the original OCP area. We have already reported on the spatial and temporal distribution of species within the *Simulium damnosum* complex found at breeding sites in southern Ghana from 1971 to 2011 [25], and on the biting and parous rates of host-seeking females [26]. In this paper, we present the spatial and temporal patterns of infection with *Onchocerca* spp. larvae of host-seeking and ovipositing flies in communities that have experienced different durations (and frequency) of ivermectin treatment. We relate our findings to the therapeutic coverage recorded in each study village and discuss the potential of fly trapping techniques, not based on the traditional OCP vector collector method, for the monitoring of transmission prior to or after the initiation of post-MDA surveillance.

## Materials and Methods

### Ethics Statement

Ethical clearance was obtained from the Imperial College Research Ethics Committee (ICREC_9_1_7) and the Institutional Review Board of the Noguchi Memorial Institute for Medical Research, University of Ghana (IRB:0001276, 006/08-09). No tissue samples were taken from human subjects; however, local villagers and elders assisted with blackfly collections. Signed informed consent was obtained from all individuals involved after detailed explanations in their local languages about the study. Participating individuals were not at an increased risk of exposure, nor were human samples obtained for diagnosis, therefore, no treatments were offered. However, all participants were receiving ivermectin as part of the national programme following appropriate (annual or biannual) schedules according to the Ghana Health Service strategy [24].

### Study Area

**Fig 1:**
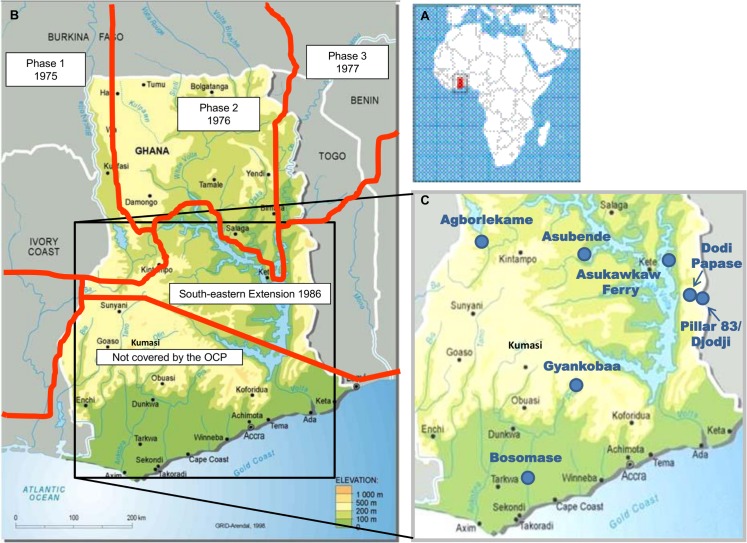
Maps showing the location of Ghana (A), the boundaries and start dates for the Onchocerciasis Control Programme (OCP) phases (B), and the seven Ghanaian study sites (C). The OCP began vector control operations across West Africa in 1975. Asubende received vector control from 1986, which was interrupted several times during 1987–1989 because of community trials of the impact of ivermectin mass treatment on transmission and microfilarial loads. At the time of closure of the OCP in 2002, the Asubende focus was incorporated into a special intervention zone (SIZ) due to on-going transmission. The breeding sites at Asukawkaw Ferry, Dodi Papase and Pillar 83 were first treated with larviciding insecticides during OCP experimental campaigns (reinvasion studies) in 1981 (see Fig 2 of Cheke & Garms 1983 [94]), before becoming part of the South-eastern extension, which reached these river basins when it became fully operational in February 1988.

Site selection, geography and key simuliid species are described elsewhere [26], but, in brief, blackfly collection was conducted in seven villages within four regions of Ghana: Asubende (08°01'01.4"N, 00°58'53.8"W) and Agborlekame (08°14'04.0"N, 2°12'23.2"W) in the Brong-Ahafo Region; Asukawkaw Ferry (07°40'55.9"N, 00°22'16.0"E), Dodi Papase (07°43'22.5"N, 00°30'38.3"E) and Pillar 83 (07°42'20.3"N, 00°35'21.5"E) in the Volta Region (Pillar 83 is the village on the Ghanaian side of the river Wawa, which forms the border and is known as the Gban-Houa in Togo, opposite the former OCP catching site of Djodji in Togo); Bosomase (05°10'44.7"N, 01°36'23.1"W) in the Western Region and Gyankobaa (06°20'12.4"N, 01°16'11.3"W) in the Ashanti Region (Fig 1). A pilot study was conducted at Bosomase in January–February 2006 to assess the efficacy of Bellec traps (see below) as a fly collection method, and to test the performance of DNA amplification methods for the determination of blackfly species, infection status and blood meal origin. The main sample collection took place during one wet season, 23^rd^ July–5^th^ September 2009, and two dry seasons, 14^th^ February–28^th^ March 2010 and 30^th^ January–5^th^ March 2011. Villages were visited and samples were collected for up to five consecutive days per site per trip. Not all sites were successfully sampled during each period due to weather conditions and variability in blackfly population abundance.

### Blackfly Sample Collection

#### Aquatic stages

Larvae and pupae were collected from submerged vegetation in fast flowing stretches of the rivers. They were preserved in Carnoy’s solution (ethanol: acetic acid, 3:1) for chromosomal identification. Chromosomal preparations and identification to species and cytoforms were carried out by standard cytotaxonomy techniques [27]. Findings have been published elsewhere [25] but they are used here to inform the morphological identifications of adult flies (see below) to assist with assessment of vector species composition.

#### Ovipositing blackflies

**Fig 2:**
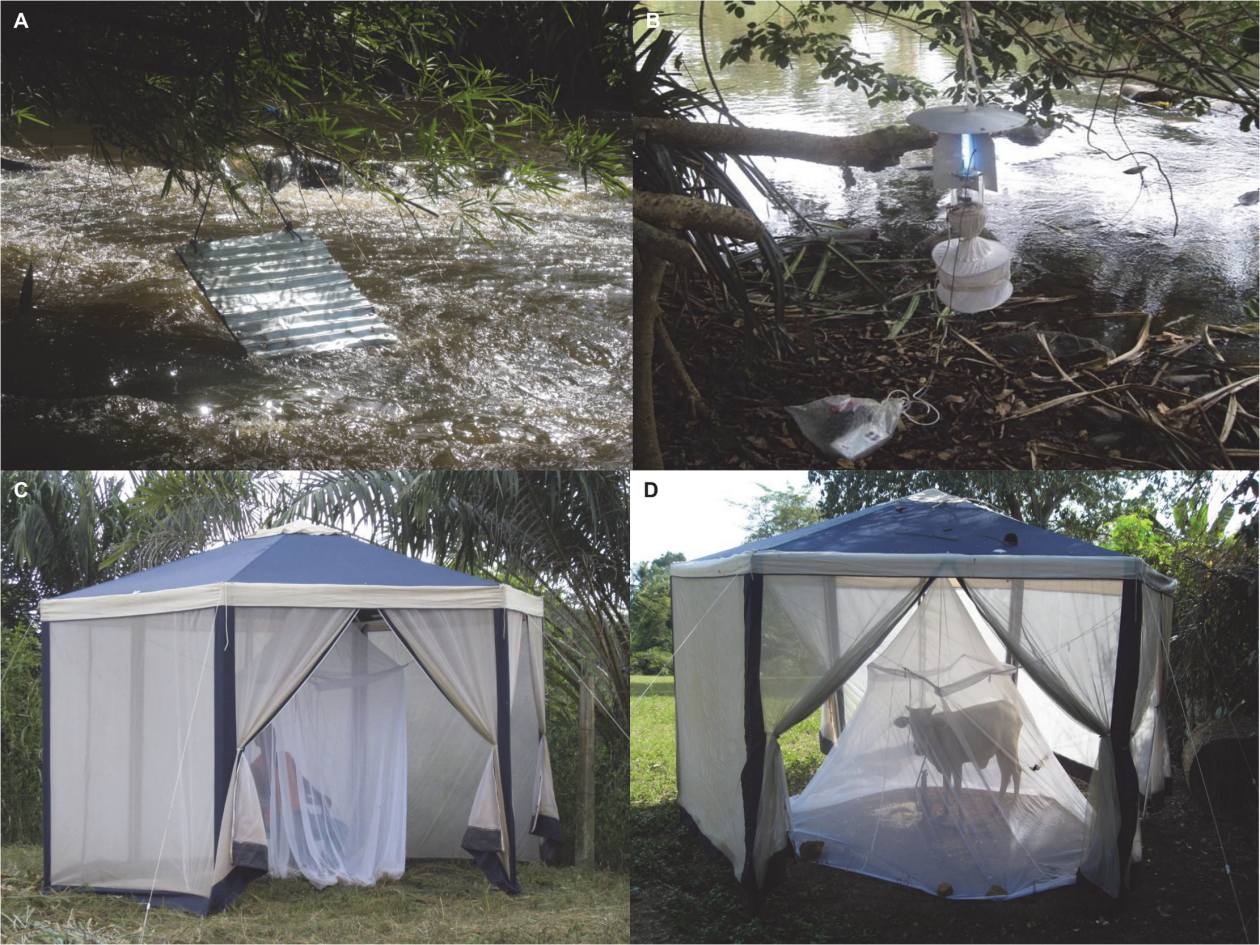
Methods used to obtain host-independent (A, B) and host-dependent (C, D) adult female blackfly samples. **(A)** Bellec (sticky) trap situated above rapids; **(B)** Monk’s Wood (light) trap placed near presumed breeding sites; **(C)** human-baited tent; **(D)** cow-baited tent. **A** and **B** illustrate traps to collect ovipositing flies; **C** and **D** depict methods to obtain host-seeking flies.

Sticky Traps (Bellec Traps): Bellec traps were set following a modified protocol based on previously proven procedures [28]. The traps consisted of corrugated iron sheets of approximately 1m^2^, with perforations to fasten floats and attach ropes, suspended from vegetation approximately one metre from rapids in the river (Fig 2A) at a 45° angle with the water surface, or on the river banks (<2m from rapids). Six traps, each at least 4m apart, were coated with coconut or peanut oil and checked at 08:00 and 17:00 daily for five days at each location per sampling trip. Blackflies stuck on the plates were collected with forceps, washed with xylene to remove the oil, counted, morphologically identified and preserved in a solution of equal parts of xylene: absolute ethanol. The traps were rinsed with water between collections before being re-coated with coconut or peanut oil. If no blackflies were collected after 24 hours, the location of the trap was changed to maximize sampling success.

Light Traps (Monk’s Wood Traps): Two UV Monk’s Wood light traps [29,30] were set approximately 20m apart, and at least 6m from any Bellec trap, at the riverside in each village, every day during each sampling period, between dusk and dawn and examined every morning (Fig 2B). Trapped insects were killed with methanol and any blackflies collected were preserved in absolute ethanol for subsequent identification. If no blackflies were collected after 24 hours, the location of the trap was changed to maximize sampling success. Cables attaching the 12V batteries on which the light traps operated were coated in grease to prevent removal of the trap contents by ants.

#### Host-seeking blackflies

Host-seeking blackfly collection has been described in detail elsewhere [26], but in summary, blackflies were caught using a human-baited (gazebo-like) tent, a cattle-baited tent (Fig 2C and 2D) and the standard OCP vector collector methods [31]. (The motivation for collecting flies attracted to cattle stemmed from the objective of the overall study, aiming to understand patterns of blood host choice by onchocerciasis vectors and their impact on transmission dynamics. Therefore, we also report here the results obtained from the cow-baited tent and pay particular attention to the species identification of *Onchocerca* larvae, a proportion of which could have been of zoonotic (cattle) origin.) The human and cattle-baited tents were set >20m apart from each other and >20m from any oviposition trap. The vector collector was >20m from any other catching trap. Blackflies were collected hourly from 07:00 to 18:00 each day. Flies were stored individually in tubes in a cool box and then refrigerated overnight before being morphologically identified and dissected for parity the following day [26].

In summary, a total of six Bellec traps, two Monk’s Wood light traps, one human-baited tent, one cattle-baited tent and one standard OCP vector collection was used every day for five days at each location per sampling trip.

### Blackfly species identification

In Ghana, six main species are known to contribute to the transmission of *O*. *volvulus*. These are *S*. *damnosum* sensu stricto (s.s.) Vajime and Dunbar; *S*. *sirbanum* Vajime and Dunbar; *S*. *sanctipauli* Vajime and Dunbar; *S*. *yahense* Vajime and Dunbar; the Beffa form of *S*. *soubrense* Vajime and Dunbar [32] and *S*. *squamosum* (Enderlein) (of which both C and E forms occur) [25]. Morphological identifications, parity status and molecular fly identifications have been described in detail previously [26] and were carried out using standard methods [32,33,34,35,36,37,38,39,40,41]. The colour of the fore-coxae used by some authors [33,34] to separate *S*. *damnosum* s.s. from *S*. *sirbanum* is unreliable since many individuals of both species with either dark or pale fore-coxae have been noted, especially in the eastern parts of the former OCP, and therefore these two species were not split by definitive identification and are termed *S*. *damnosum* s.s. /*S*. *sirbanum*. Morphological identifications and parity status of the host-seeking blackflies were performed the day after being caught. Parous females’ abdomens were separated from the head and thorax, which were preserved individually in corresponding wells of two 96-well PCR plates (one for heads plus thoraces, one for abdomens) in absolute ethanol for subsequent molecular analysis. When catch numbers were manageable (up to 300 flies per day), all host-seeking flies were first dissected for parity in the field. When parity of some blackflies was not assessed due to high catch numbers and time constraints (>300 per day), all remaining host-seeking flies were only morphologically identified and their heads and thoraces separated from their abdomens and stored as above. *Simulium squamosum* shares many morphological traits with other sympatric species, causing difficulties when identifying some adult blackflies [33]. Therefore, DNA from all abdomens was extracted and used for definitive molecular identification of *S*. *squamosum* and for *Onchocerca* spp. infections as described below. Flies caught in Bellec and Monk’s Wood traps were morphologically identified using the same techniques [35,36,37,38,39,40,41], and the heads, thoraces and abdomens separated and stored individually as for the host-seeking flies [26].

### Assessment of Infection Rates

The heads and thoraces of all the known parous and unknown parous (physiological age not determined) host-seeking blackflies were dissected for *Onchocerca* infection. Flies caught in Bellec and Monk’s Wood traps were in the process of ovipositing and hence were not dissected for parity, as their gravid status made parity assessment impossible without counting their ova [42]. Although the flies coming to lay eggs in breeding sites would comprise both nulliparous (laying eggs for the first time) and parous flies (having laid eggs before), it was assumed that they would have all taken at least one blood meal (as *S*. *damnosum* s.l. is obligatorily anautogenous [43]) and, therefore, capable of ingesting *Onchocerca* microfilariae if feeding on infected hosts 2–3 days previously. By the time of oviposition, some of these microfilariae could have migrated out of the abdomen and established in the thorax as L1 larvae. In parous flies, infections picked up 2–3 gonotrophic cycles earlier, could have developed into pre-infective (L2) in the thorax, or infective (L3) larvae, found in heads or thoraces. Therefore, the heads and thoraces of all ovipositing flies were dissected for infection with *Onchocerca* larvae. Heads and thoraces were soaked in distilled water for one hour, stained with a solution of 7% lactopropionic orcein in distilled water for a further hour [44], and examined in a drop of the staining solution under a dissecting microscope. The numbers, developmental stage (L1, L2, L3), and location within the fly (head or thorax) of any *Onchocerca* spp. larvae were recorded. Larvae were transferred to steel-frame 0.9μm POL-membrane slides (Microdissect, Leica, Germany) [45] for subsequent individual DNA-based identification of parasite species (such as *O*. *volvulus*, *O*. *ochengi*, *O*. *ramachandrini*, *O*. *dukei*, *O*. *denkei* and the Siisa-clade of *O*. *ochengi*) [46,47,48]. In the field, during the morphological identification and parity dissection, any *Onchocerca* larvae which emerged were also recorded and transferred to a POL-membrane slide.

### Molecular Analysis of *Onchocerca* Species

Since *S*. *damnosum* s.l. is also involved in the transmission of other *Onchocerca* species [46,49], parasite larvae were identified by molecular methods to ensure that transmission of human onchocerciasis would be accurately recorded. POL-membrane slides with the *Onchocerca* spp. L1, L2 and/or L3 were placed on a Leica LMD6000 laser dissection microscope, viewed on a computer screen, and any *Onchocerca* larvae were cut out individually using an ultraviolet laser, with the sample falling into a PCR tube cap below [45]. Larvae were stored in 15μl Qiagen ATL buffer and frozen until DNA extraction. DNA extraction was performed using the QIAamp DNA Micro kit (QIAGEN) following the ‘isolation of genomic DNA from laser-microdissected tissues’ protocol, with DNA eluted into 30μl sterile distilled water. DNA was amplified using general *Onchocerca* (primer O-150) [47,50] and the *O*. *volvulus* specific (C1A1-2) [47] primers and the results run on agarose gels for species identification through presence or absence of the *O*. *volvulus* specific amplicon, when the *Onchocerca* general PCR had been successful. In addition, PCR amplifications were performed using three further pairs of primers 12SOvB and C, 16SOvB and C, and ND5OvA and C amplifying 12S rRNA, 16S rRNA, and ND5 mitochondrial genes respectively [51,52]. PCR clean-up, quantification and sequencing was performed on these 12S, 16S, and ND5 amplicons. Sequences were then individually run through BLAST and *Onchocerca* species identification scored when successful matches occurred. Sequences were also compared to known sequences of *Onchocerca* on ClustalW for additional clarification of any species identification. PCR plates contained negative water controls, *O*. *ochengi* (adult worm DNA) positive controls, and *O*. *volvulus* (microfilarial DNA) positive controls. Presence of *Onchocerca* (most likely microfilariae or infective larvae) in the abdomens was detected using the same 16S protocol [51] mentioned above for dissected *Onchocerca* larvae; any positive amplicons were then also sequenced and run through BLAST and ClustalW.

### Treatment History

The study communities currently receive community-directed treatment with ivermectin (CDTI) but with varying treatment histories in terms of number of years of MDA and treatment frequency, as well as having experienced a range of historical vector control activities, summarised in Table 1. Community drug distributors were interviewed regarding recent drug administrations in each village, as well as village, regional and national treatment records checked for historical treatments. Dates of historical vector control are indicated in Fig 1 and previously discussed in [26]. Data on yearly therapeutic coverage of ivermectin for each study village for annual or biannual treatment rounds were provided by the Ghana Health Service.

**Table 1:** Vector control and ivermectin treatment history, current strategy and infection levels per village.

Region	Village	Vector control history	First ivermectin treatment round	Ivermectin treatment frequency and year(s) it applies from	Baseline year, microfilarial prevalence in % (CMFL^a^)	Year of CDTI^b^ missed since 2000	Date of last treatment prior to data collection	Start date of data collection (season)	Percentage of flies infected^c^ (95%CI)	Percentage of flies infective^d^ (95%CI)
**Brong-Ahafo**	**Asubende**	1986–2002 (interruptedduring 1987–1989 for ivermectin trials)	1987	Annual 1987–2009, Bi-Annual since 2009	1980, 76.1% (23.8mf/ss)	2005	Dec 2010	07/02/11 (Dry)	0.1 (0.0–0.5)	0.1 (0.0–0.5)
** **	**Agborlekame**	1975–2002	1987	Annual 1987–2009, Bi-Annual since 2009	1980, 66.5% (23.3mf/ss)	2005	Jan 2010	21/02/10 (Dry)	0.0 (0.0–2.3)	0.0 (0.0–2.3)
**Volta**	**Asukawkaw Ferry**	1981, 1988–2002	1993	Annual since 1993	1978, 76.0% (14.3mf/ss)	2005	Jan 2008	03/08/09 (Wet)	0.0 (0.0–1.0)	0.0 (0.0–1.0)
** **							Jan 2010	16/03/10 (Dry)	0.1 (0.0–0.5)	0.0 (0.0–0.4)
** **	** **						Dec 2010	24/02/11 (Dry)	0.0 (0.0–0.4)	0.0 (0.0–0.4)
** **	**Dodi Papase**	1981, 1998–2002	1993	Annual since 1993	1978, 66.8% (16.8mf/ss)	2006	Jan 2009	09/08/09 (Wet)	0.0 (0.0–1.6)	0.0 (0.0–1.6)
** **	** **						Jan 2010	11/03/10 (Dry)	0.0 (0.0–1.0)	0.0 (0.0–1.0)
** **							Dec 2010	21/02/11 (Dry)	0.0 (0.0–0.6)	0.0 (0.0–0.6)
** **	**Pillar 83/ Djodji**	1981, 1988–2002	1993	Multiple 1993–1997, Annual since 1998	2000, 6.8%^**e**^ (0.09mf/ss)	2001, 2005	Feb 2009	28/07/09 (Wet)	0.0 (0.0–7.6)	0.0 (0.0–7.6)
** **							Jan 2010	06/03/10 (Dry)	0.0 (0.0–0.2)	0.0 (0.0–0.2)
** **	** **						Dec 2010	17/02/11 (Dry)	0.0 (0.0–0.1)	0.0 (0.0–0.1)
**Western**	**Bosomase**	None	2003	Annual since 2003	2002, 41.0% (1.35mf/ss)	2007	Jun 2008	19/08/09 (Wet)	1.4 (0.2–2.5)	0.7 (0.1–1.6)
** **	** **						Jan 2010	24/02/10 (Dry)	1.9 (0.3–3.2)	1.1 (0.2–2.1)
**Ashanti**	**Gyankobaa**	None	2006	Annual 2006–2009, Bi-Annual since 2009	2006, 45.1% (2.89mf/ss)	None	Sept 2008	26/08/09 (Wet)	2.1 (0.4–2.8)	1.1 (0.2–1.6)
** **	** **						Dec 2009	17/02/10 (Dry)	0.0 (0.0–30.9)	0.0 (0.0–30.9)

^**a**^ CMFL = community microfilarial load as defined in [19], expressed as microfilariae (mf) per skin snip (ss)

^**b**^ CDTI = community-directed treatment with ivermectin

^**c**^ Flies infected with any *O*. *volvulus* larval stage

^**d**^ Flies infected with *O*. *volvulus* L3 larvae in heads and thoraces (the percentages of infected and infective flies were calculated from all flies, collected by both oviposition and host-seeking methods. Details of the total numbers dissected for each collection technique per village per season are presented in S2 Table)

^**e**^ pre-treatment baseline prevalence unknown, with 2000 the earliest date available; multiple ivermectin treatments from 1993 to 1997. In 1992, a mapping survey of the Onchocerciasis Control Programme in West Africa extension stated that ‘the site of Djodji presents the highest transmission potentials of the Eastern extension’[95].

### Statistical Analyses

Except where specified as PCR results on the blackfly abdomens, all data presented are from dissections of heads and thoraces only. Data are reported as per fly, per parous fly, per infected fly or infective fly throughout. The **proportion infected** is taken as the number of flies of each species with any larval stage (L1, L2 or L3) divided by the total number of flies of that species dissected and are presented with 95% exact confidence intervals (95% CI), determined using the method of Clopper-Pearson [53].

Because *Onchocerca* L3s can migrate from other parts of the body to the head during a blood meal, a fly with L3s in any body part is counted as infective [54,55,56]. (Infective larvae develop in the fly’s thoracic muscles and typically migrate to the head capsule and the fly’s proboscis, but they have also been detected in the halteres and abdomen.) Therefore, the **proportion infective** is the number of flies of each species with L3 larvae (in head and/or thorax) divided by the total number of flies of that species dissected and is presented with 95% CIs. In addition we also present the number of flies with L3s in the head only for comparison with published literature.

We calculated monthly infective biting rates, which take into account the number of infective flies that (come to) bite a host per month, but not their parasite burden. These were calculated by multiplying the proportion of infective flies (with L3 larvae in head and/or thorax) by the monthly biting (landing) rates as reported elsewhere [26], but summarised in S1 Table. Monthly parous biting rates, the monthly rate at which a host would be bitten by parous flies, have been presented and analysed by species elsewhere [26].

We calculated the arithmetic mean number of L3 per infective fly per species (**L3s/infective fly**) as the total number of L3 larvae divided by the number of flies which contained any L3 larvae. The monthly transmission potential is the mean number of L3 larvae to which a host is exposed per month. These were calculated by multiplying monthly infective biting rates by the number of L3s/infective fly. We report transmission potentials for given months in the wet and dry seasons, but as we did not collect data throughout the whole year we do not extrapolate these results to annual transmission potentials. As fly survival rates have been shown to affect variations in transmission rates [57,58], we also present the number of L3 larvae per 1,000 parous flies as recommended by the WHO [11]. These values are reported, separately, for parous host-seeking flies and ovipositing flies for each location and season. The mean number of **L3s/1,000 parous (or ovipositing) flies** was calculated as the total number of L3 larvae divided by the total number of parous (or ovipositing) flies dissected for *Onchocerca* multiplied by 1,000. We did not assume that the same parity rates determined in samples of host-seeking flies would apply to the ovipositing flies caught near (by light traps) or in breeding sites (by Bellec traps) because a phenomenon of differential dispersal of nulliparous and parous flies along rivers and inland from rivers has been documented in *S*. *damnosum* s.l., which varies between the savannah and forest members of the species complex [59].

The transmission indices described above were calculated from flies captured by vector collectors (and therefore relate to human exposure and the potential of transmission from flies to humans) unless stated otherwise. Host-seeking infective flies collected in the cow tents—had they been able to bite cattle and shed their entire L3 larval load—would not have contributed effectively to the transmission of *O*. *volvulus*. However, these flies indicate occurrence of active transmission from humans to flies, as they have become infected and survived the incubation period of the parasite. Therefore, these transmission parameters are presented for each host-seeking catching technique. Also, our results indicate that flies that bite cattle may also bite humans (blood meal results to be presented elsewhere) and so, if able to survive further gonotrophic cycles, infected and infective flies attracted to cattle could subsequently feed on humans and transmit their remaining infective larval load as, on average, only 50 to 80% of L3 larvae are shed per bite [55,60]. The proportion infected, proportion infective, the mean number of L3s/infective fly and the number of L3s/1,000 (parous or ovipositing) flies are reported, separately, for host-seeking and ovipositing flies.

Statistical analyses were performed on SPSS version 22 (SPSS, Inc., Chicago, IL, USA) or R [61]. Numbers of infected and infective flies, for all catches, and per species, were compared among villages, seasons and trapping methods using chi-squared (χ^2^) tests. Ninety five percent CIs for the number of L3/1,000 parous, L3 per 1,000 ovipositing and L3 per infective flies were determined using a percentile bootstrap method [62]. A correlation between the number of years since the start of ivermectin treatment and the proportion of infected and infective flies was tested using Spearman’s Rank correlation coefficient (*r*_*S*_). Variation in infection intensities among different species was compared using Kruskal Wallis and Mann–Whitney U tests. Numbers of infected versus uninfected flies as measured by PCR of the abdomens were compared between catching techniques using the chi-squared (χ^2^) test. Therapeutic coverage of ivermectin distribution was plotted against time since each village commenced treatment, with a best fit polynomial plotted for each village.

## Results

A total of 17,300 *S*. *damnosum* s.l. flies was collected, of which 6,142 (35.5%) were caught by vector collectors; 2,207 (12.8%) were trapped in the human-baited tents; 1,567 (9.1%) in the cow-baited tents; 7,212 (41.7%) on Bellec traps—including 3,352 (46.5% of the Bellec total) from the pilot study in Bosomase during the dry season in 2006—and 172 (1%) in Monk’s Wood light traps. A total of 16,478 (95.2%) blackflies was morphologically identified, of which 5,812 (35.3%) were dissected for parity in the field, with 4,247 (73.1%) nullipars and 1,565 (26.9%) parous flies. These nullipars were not further dissected for *Onchocerca* infection, but pooled samples of the nullipars were used as molecular controls, with no positive *Onchocerca* results obtained. The heads and thoraces of 12,196 flies (6,918 ovipositing flies, 3,713 host-seeking flies of unknown parity status and 1,565 known parous flies) were stained and dissected for *Onchocerca* spp. larvae. These, plus the known uninfected nullipars (4,247), totalled 16,443 flies whose infection status was assessed. A total of 97 (0.6%) was infected (with any larval stage) of which 58 (0.4%) were infective (with L3s in head and/or thorax), with 45 flies (0.3%) harbouring L3s in the head (S2 Table).

### *Onchocerca* spp. Identification

DNA was extracted and amplified from all 463 larvae of all stages, from the 97 infected flies (on average, 4.8 larvae per infected fly and 0.03 per fly). The PCR product using the ND5 primers was consistently of poor quality and therefore only the 12S and 16S amplicons [51] were used for *Onchocerca* spp. identification with BLAST and ClustalW. Of all individual L1 to L3 larvae, 76% (352/463) were positively identified as *O*. *volvulus* using either 12SOv, 16SOv primers and/or *O*. *volvulus* specific (O-150 versus C1A1-2) amplicons in the agarose gels. The remaining 24% were not successfully amplified. No *O*. *ochengi* was observed in the field-caught flies, but the positive *O*. *ochengi* controls were successfully identified by BLAST and/or ClustalW and did not have *O*. *volvulus* specific amplicons in the agarose gels. There were no ambiguous results for the species identification. Of the 111 non-identifiable larvae, 107 (96%) came from flies in which other larvae of the same stage had been successfully identified as *O*. *volvulus*.

### *Onchocerca volvulus* Transmission

Blackflies infected with *O*. *volvulus* larvae were recorded at Asubende, Asukawkaw Ferry, Bosomase and Gyankobaa, and infective flies (with L3s in head and/or thorax) were recorded at Asubende, Bosomase and Gyankobaa (Tables 1 and S2). No infected or infective flies were observed at Agborlekame, Dodi Papase or Pillar 83 during our study from the heads and thoraces; however, *O*. *volvulus* DNA was amplified in flies from all seven villages from the abdomens (see below).

**Fig 3:**
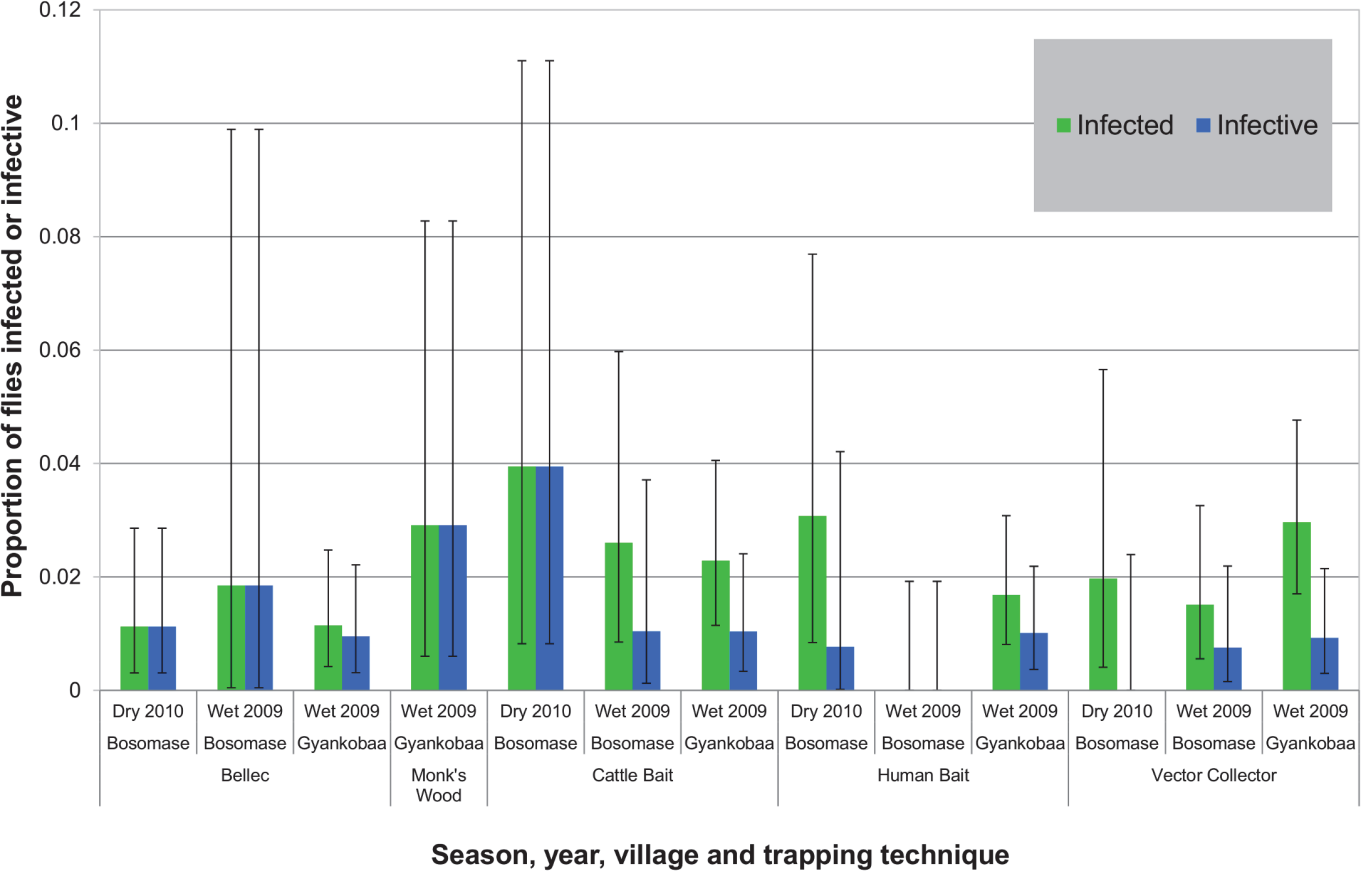
Proportions of infected and infective flies by season and sampling method for Bosomase and Gyankobaa. Data and error bars are as in Fig 3, but excluding the 2006 Bellec-caught flies collected at Bosomase, as during the pilot study comparisons with other fly collection methods were not conducted.

There was no statistically significant difference in the proportion of infected (χ^2^ = 5.06, d.f. = 3, *p* = 0.168) and infective (χ^2^ = 2.79, d.f. = 3, *p* = 0.425) flies caught at Gyankobaa or Bosomase by the different trapping methods. A higher but, not statistically significant, proportion of infected and infective parous flies were caught in the cow-baited tents (infected = 2.54%, infective = 1.34%) than the other trapping methods (Fig 3), with infected and infective levels of 1.53% and 0.77% in the human-baited tents, 2.30% and 0.73% by the vector collectors and 1.06% and 0.97% in the oviposition traps, respectively. Twenty seven percent of the infected flies were caught in the cow-baited tents, 19% in the human-baited tents, 34% in the vector collector caught flies and 20% by the oviposition traps.

There was no statistically significant difference between the proportion of infected (χ^2^ = 0.90, d.f. = 1, *p* = 0.353) or infective (χ^2^ = 2.09, d.f. = 1, *p* = 0.148) flies caught by the oviposition and host-seeking methods combined, nor between the two most successful catching techniques, namely the Bellec traps and the vector collector method (infected: χ^2^ = 3.08, d.f. = 1, *p* = 0.079; infective: χ^2^ = 0.30, d.f. = 1, *p* = 0.584). In contrast, in the abdomens, statistically significantly more flies had *O*. *volvulus* infections, as recorded by PCR, in the ovipositing flies than in the host-seeking flies (χ^2^ = 19.58, d.f. = 1, *p*<0.001), as well as in just the Bellec-caught flies in comparison with the vector collector-caught flies (χ^2^ = 8.51, d.f. = 1, *p* = 0.004).

**Fig 4:**
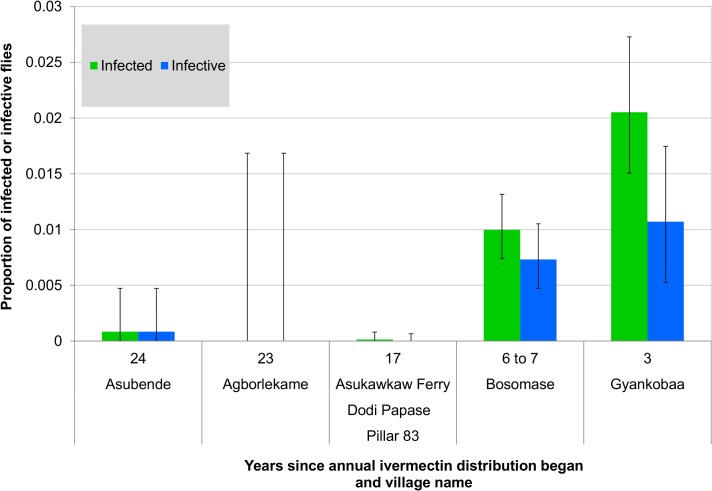
Proportions of infected and infective flies (assessed by heads and thoraces dissection) in study villages and years of ivermectin treatment. Infected flies (green bars) are those with any larval stage of *Onchocerca volvulus*; infective flies (blue bars) are those harbouring L3 larvae in heads and/or thoraces. Error bars are exact 95% confidence intervals. The results for Bosomase include the Bellec-caught flies obtained during the pilot study conducted at Bosomase in January–February 2006.

There was a negative correlation between the number of years since the start of ivermectin treatment and the proportion of infected and infective flies as measured from all those dissected, including the nullipars (Table 1) (infected: *r*_*s*_ = –0.717, *p* = 0.045; infective: *r*_*s*_ = –0.654, *p* = 0.078) and parous flies (infected: *r*_*s*_ = –0.700, *p* = 0.188; infective: *r*_*s*_ = –0.667, *p* = 0.219) (Fig 4), but this reached statistical significance only for the overall proportion infected.

**Fig 5:**
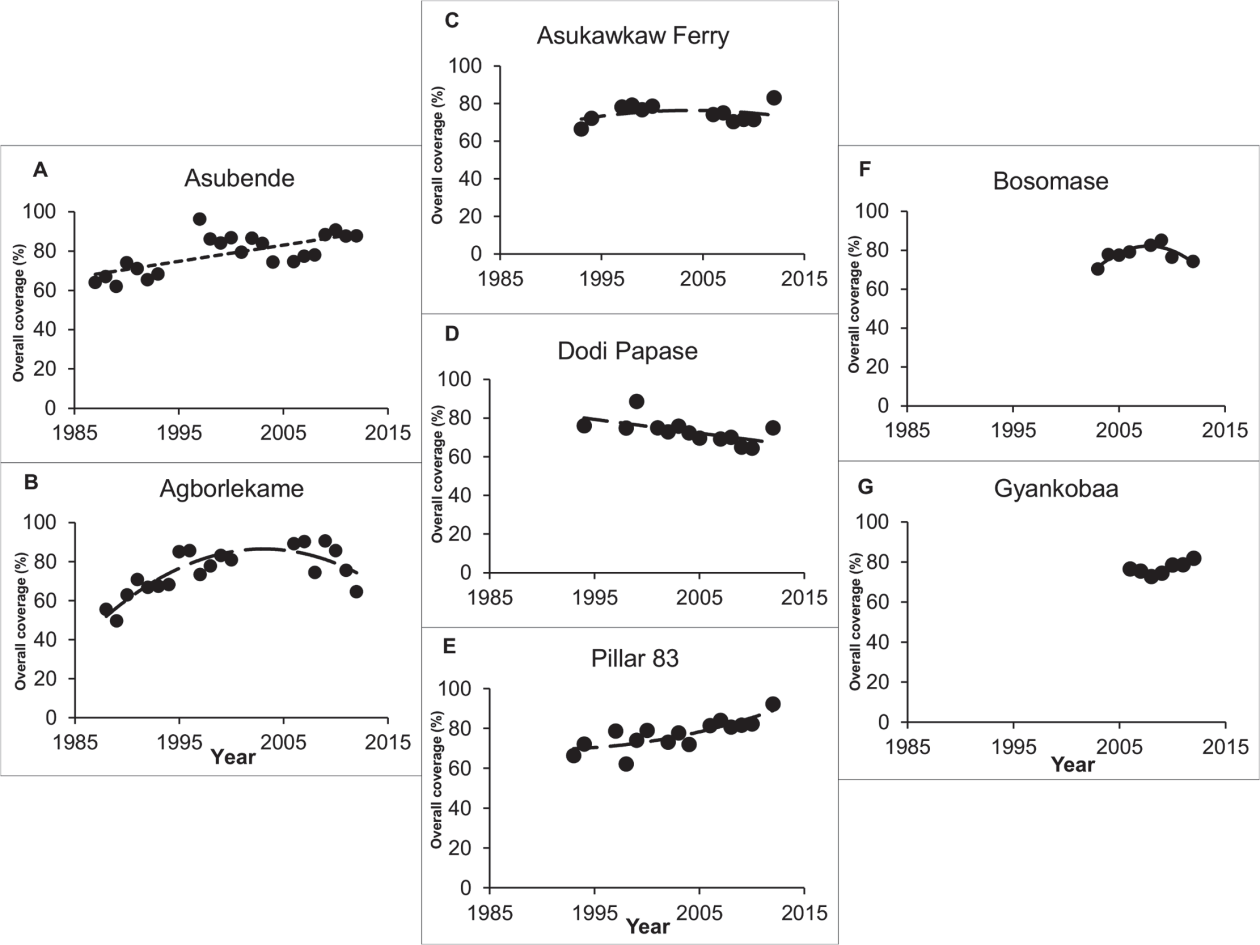
Therapeutic coverage of ivermectin treatment in all study villages. The plots show the percentage of the overall population treated at each ivermectin round since mass ivermectin distribution began: **(A)** Asubende and **(B)** Agborlekame in the Brong-Ahafo Region; **(C)** Asukawkaw Ferry, **(D)** Dodi Papase and **(E)** Pillar 83 in the Volta Region; **(F)** Bosomase in the Western Region, and **(G)** Gyankobaa in the Ashanti Region. The dashed lines are the best fit least squares polynomial functions to the data, presented to facilitate visual inspection of the coverage trends. Biannual ivermectin distribution started in 2009 in Asubende, Agborlekame and Gyankobaa, whilst annual distribution has continued in the remaining villages.

Therapeutic coverage (the proportion of the overall population treated with ivermectin) for all villages was rarely below 60%. Coverage in Asubende, Pillar 83 and Gyankobaa had steadily increased since the beginning of mass treatment implementation, whilst Agborlekame, Dodi Papase, and Bosomase appeared to experience a recent decreasing trend in treatment coverage (Fig 5).

### Human Exposure

Monthly infective biting rates and monthly transmission potentials calculated from host-seeking flies only were zero in all villages except for Bosomase and Gyankobaa, the villages most recently incorporated into the CDTI programme. These transmission indices were also negative for Asubende, as the only fly identified as infective was an ovipositing blackfly caught using a Bellec trap, rather than a host-seeking fly.

Monthly infective biting rates varied greatly between villages, seasons, catching techniques and vector species (Table 2). In Bosomase, for human and cattle-seeking catching methods, these rates ranged from 0 to 42.2 infective bites/host/month, with higher values in the wet season than in the dry season. In the wet season of 2009, the forest form of *S*. *sanctipauli* was the main vector recorded in Bosomase and the only species with infective larvae, whilst in the dry season of 2010, *S*. *yahense* was the main vector species harbouring infective larvae (Table 2). At Gyankobaa, only 11 flies were collected in the dry seasons of 2010 and 2011 (S2 Table), all from Bellec traps, but in the wet season of 2009, the infective biting rates ranged from 38.9 infective bites/person/month, caught by vector collectors, to 50.4 infective bites/cow/month, for flies collected in the cow-baited tents (Table 2). *Simulium sanctipauli* flies harboured infective larval stages across all catching techniques at Gyankobaa indicating that this species was able to pick up infections from humans (although they would later attempt to feed on a non-human host), whereas infective *S*. *damnosum* s.s./*S*. *sirbanum* were only caught in the man-baited tents or by vector collectors, contributing both to transmission from humans to flies and from flies to humans. However, the overall sample sizes of *S*. *damnosum* s.s./*S*. *sirbanum* at Gyankobaa from the wet season of 2009 were low, with only 35, 4 and 13 *S*. *damnosum* s.s./*S*. *sirbanum* caught and dissected for infection from the vector collectors, human-baited and cow-baited tents, respectively.

**Table 2:** Monthly Infective Biting Rates (MIBRs) of host-seeking blackflies by locality, season, trapping technique and species.

Region	Village	Season	Trapping Method	*S*.* damnosum* s.l. (average)	*S*.* damnosum* s.s. */S*.* sirbanum*	*S*.* soubrense* Beffa form	*S*.* squamosum*	*S*.* yahense*	*S*.* sanctipauli*
Western	Bosomase	WetAug 2009	V/C	42.21	-	-	-	-	42.21
			Human-tent	0	-	-	0	-	0
			Cow-tent	17.22	-	-	-	-	17.22
			Host-seeking	18.70	-	-	0	-	18.70
		Dry Feb 2010	V/C	0	-	-	-	0	0
			Human-tent	6.47	-	-	-	0	7.14
			Cow-tent	18.43	-	-	-	16.98	0
			Host-seeking	7.36	-	-	-	4.99	2.48
Ashanti	Gyankobaa	WetAug 2009	V/C	38.89	7.53	-	0	0	28.60
			Human-tent	42.82	8.50	-	0	0	34.30
			Cow-tent	50.44	0	-	0	0	51.09
			Host-seeking	43.99	6.80	-	0	0	37.17

V/C = vector collector.

The values for V/C and Human-tent indicate infective bites per person per month; the values for Cow-tent are infective bites per cow per month; the values for Host-seeking are the average values per host per month.

Although flies harbouring *O*. *volvulus* were caught at both Asubende and Asukawkaw Ferry, the infected/infective *S*. *damnosum s*.*s*.*/S*. *sirbanum* fly at Asubende was ovipositing and not host-seeking and therefore does not contribute to MIBR; the *S*. *soubrense* Beffa form fly at Asukawkaw Ferry was infected but not infective.

**Fig 6:**
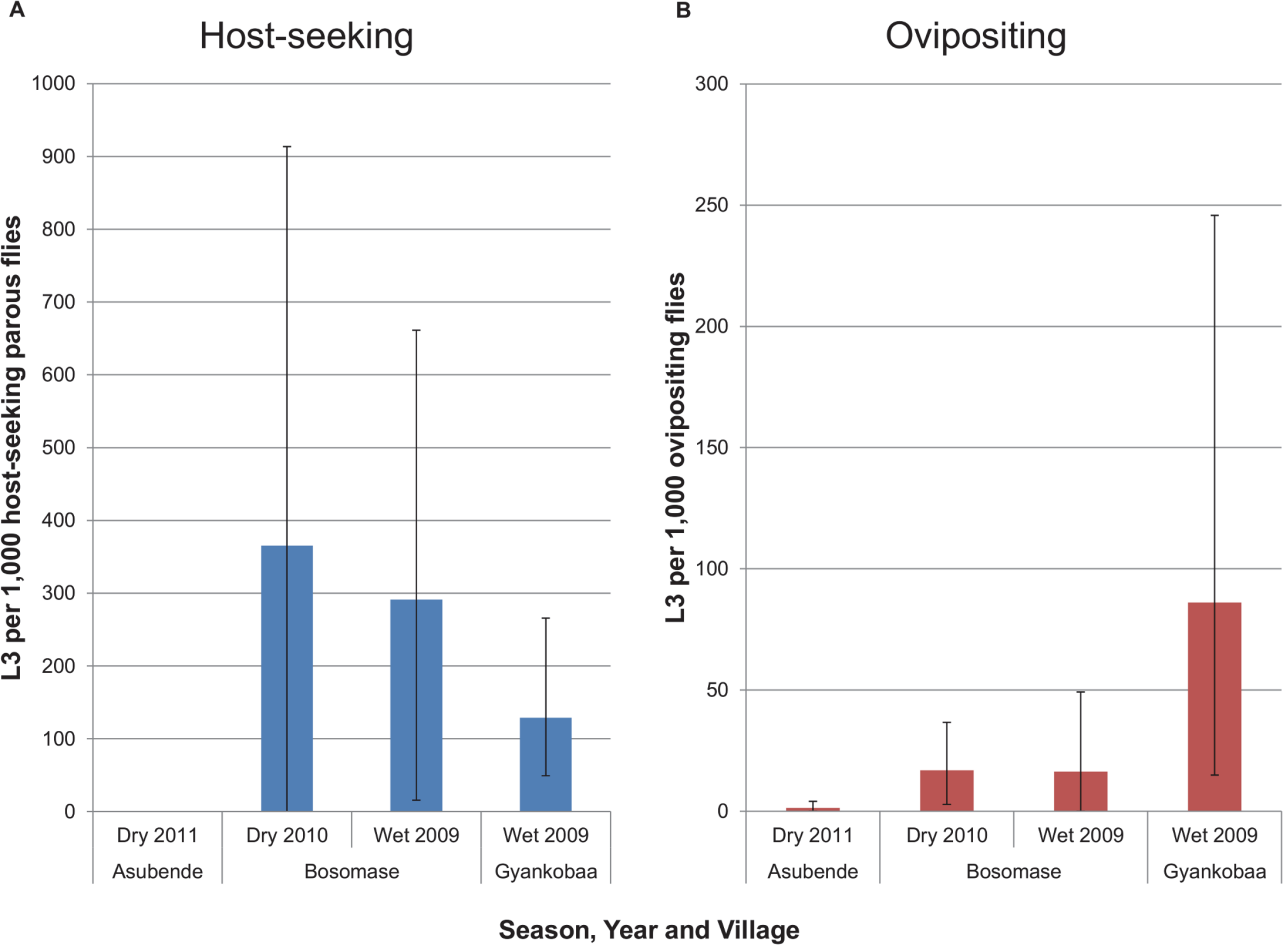
Numbers of L3 larvae per 1,000 parous (host-seeking) flies or per 1,000 ovipositing flies. Data are presented for the three villages where infective *Onchocerca volvulus* larvae were detected during the wet and/or dry seasons of 2009 to 2011. In all cases, except Asubende parous flies, the number of (A) *O*. *volvulus* L3 larvae per 1,000 parous or (B) *O*. *volvulus* L3 larvae per 1,000 ovipositing flies exceeds the World Health Organization (WHO) threshold for one L3 per 1,000 (parous) flies. In Asubende the value was 1.4 L3 per 1,000 ovipositing flies. Error bars are 95% CI calculated using percentile bootstrapping.

The number of L3 larvae recorded varied between villages, seasons and catching techniques (S2 Table). The WHO states that a level of less than one L3 per 1,000 parous flies is required to control onchocerciasis transmission [11]. Gyankobaa in the wet season had levels of over 100 L3s per 1,000 parous flies whilst at Bosomase in the dry and wet season these were more than 350 and 250 L3s per 1,000 parous flies respectively (Fig 6A). Both these villages had not been included in the former OCP and were incorporated into the CDTI programme more recently in 2006 for Gyankobaa and 2003 for Bosomase. Asubende was just above this level, with 1.35 L3/1,000 (95% CI: 0–4.0) ovipositing (of which not all would be parous) flies (Fig 6B). This is despite 24 years of ivermectin treatment at the time of sampling, but the infection leading to this result was detected in an ovipositing rather than in a host-seeking fly, with 0 L3/1,000 host-seeking parous flies.

Combining L3 numbers and infective biting rates for the different vector species across trapping techniques for Gyankobaa and Bosomase resulted in transmission potentials ranging from 0 to 422.1 L3/host/month (Table 3). All flies at Asubende were *S*. *damnosum* s.s./*S*. *sirbanum*, but at Bosomase and Gyankobaa vector composition varied between seasons and catching techniques (S1 Table) [26]. *Simulium sanctipauli* was the most important vector species at both Bosomase and Gyankobaa in the wet seasons, whilst *S*. *yahense* played a more important role in transmission at Bosomase in the dry season of 2010. The importance of vector species at Bosomase in the dry season also differed between catching techniques, with *S*. *sanctipauli* having higher transmission potentials by flies caught in the human-baited tents, and *S*. *yahense* having higher transmission potentials by flies caught in the cow-baited tents (Table 3).

**Table 3:** Monthly Transmission Potentials (MTPs) of host-seeking blackflies by locality, season, trapping technique and species.

Region	Village	Season	Trapping Method	*S*.* damnosum* s.l. (average)	*S*.* damnosum* s.s. */S*.* sirbanum*	*S*.* soubrense* Beffa form	*S*.* squamosum*	*S*.* yahense*	*S*.* sanctipauli*
**Western**	**Bosomase**	**Wet**	**V/C**	422.11	-	-	-	-	422.11
		**Aug 2009**	**Human-tent**	0	-	-	0	-	0
			**Cow-tent**	60.27	-	-	-	-	60.27
			**Host-seeking**	138.36	-	-	0	-	138.36
		**Dry**	**V/C**	0	-	-	-	0	0
		**Feb 2010**	**Human-tent**	6.47	-	-	-	0	7.14
			**Cow-tent**	340.98	-	-	-	314.00	0
			**Host-seeking**	93.25	-	-	-	92.24	2.48
**Ashanti**	**Gyankobaa**	**Wet**	**V/C**	85.57	15.06	-	0	0	64.30
		**Aug 2009**	**Human-tent**	236.62	8.50	-	0	0	222.95
			**Cow-tent**	141.25	0	-	0	0	143.04
			**Host-seeking**	152.49	10.20	-	0	0	140.12

V/C = vector collector.

The values for V/C and Human-tent indicate no. L3 per person per month; the values for Cow-tent are L3 per cow per month; the values for Host-seeking are the average values per host per month.

Although flies harbouring *O*. *volvulus* were caught at both Asubende and Asukawkaw Ferry, the infected/infective *S*. *damnosum s*.*s*.*/S*. *sirbanum* fly at Asubende was ovipositing and not host-seeking and therefore does not contribute to MTP; the *S*. *soubrense* Beffa form fly at Asukawkaw Ferry was infected but not infective.

For all infected flies successfully identified to species (95 out of 97), the arithmetic mean number of *O*. *volvulus* larvae per infected fly varied greatly and statistically significantly among species, with *S*. *damnosum* s.s./*S*. *sirbanum* harbouring 1.33 larvae per infected fly ± 0.33 SE; *S*. *sanctipauli* 3.61 ± 0.40 and *S*. *yahense* 17.86 ± 4.32 (Kruskal Wallis χ^2^ = 15.50, d.f. = 2, *p*<0.001). The mean number of L3s per infective fly also differed statistically significantly among vector species, with *S*. *damnosum* s.s./*S*. *sirbanum* harbouring 1.33 L3s per infective fly ± 0.33 SE; *S*. *sanctipauli* 2.73 ± 0.50 and *S*. *yahense* 17.67 ± 9.23 (Kruskal Wallis χ^2^ = 6.83, d.f. = 2, *p* = 0.033). These differences were also observed when analysed at the village level, controlling for variations in local transmission levels, with *S*. *yahense* having significantly higher infection intensities at Bosomase in the infected flies (Mann Whitney U = 8.50, d.f. = 46, *p*<0.001). The difference had only borderline significance in the infective flies (U = 0.00, d.f. = 33, *p* = 0.057), as there was only one infective *S*. *yahense* with 17 L3s, despite the large difference between this and the mean in *S*. *sanctipauli* of 2.12 ± 0.56 L3/infective fly.

### *Onchocerca* Infections in Abdomens

Overall, 258 of the 11,122 (2.3%) abdomens tested for *Onchocerca* infections were positive. The majority of these (240) were from Gyankobaa or Bosomase; however, there was also one positive result from each of Agborlekame, Dodi Papase and Pillar 83, which had been negative by dissection of heads and thoraces. The number of infected abdomens in vector collector flies was lower (0.8%) than that in the flies caught using all other methods combined (2.3%, χ^2^ = 58.0, d.f. = 1, *p*<0.001), suggesting that the infections did not originate from the flies acquiring an infectious blood meal with microfilariae at the point of collection.

## Discussion

As the goals of onchocerciasis control programmes shift from morbidity reduction towards elimination, knowledge of ongoing transmission by local vector species is urgently required [8,9,10]. This enables entomological monitoring of programmes’ progress, and helps to understand the determinants of persistent transmission despite prolonged control interventions. We report *O*. *volvulus* transmission, in Ghanaian communities with different treatment and control histories, and its variation according to simuliid species composition, vector trapping technique and season.

Factors influencing the feasibility of achieving elimination with the current ivermectin treatment strategy include baseline levels of endemicity, patterns of treatment coverage and compliance, parasite ivermectin susceptibility, duration and effectiveness of former vector control, seasonality of transmission in relation to ivermectin distribution, parasite immigration in flies or people, vector species mix and their associated vectorial capacity and competence for *O*. *volvulus* [63].

### Potential for Infection from Humans to Flies and Flies to Humans

We have documented active onchocerciasis transmission, raising questions regarding the potential for CDTI alone to interrupt transmission under the treatment frequency and coverage levels commonly achieved in Africa. We report high monthly infectious biting rates and transmission potentials (measuring transmission from vectors to humans) for the communities most recently incorporated into the CDTI strategy. We also report infections in fly abdomens from all study villages, providing evidence of transmission from humans to flies. These infections were identified molecularly as *O*. *volvulus*. Infection levels above the WHO threshold of one L3 larva per 1,000 parous flies were recorded in the villages of Bosomase and Gyankobaa which started receiving treatment, respectively, in 2003 and 2006, i.e. 6 and 3 years prior to our entomological study. The WHO’s value forms part of the criteria for achieving the operational elimination thresholds for treatment cessation and commencement of surveillance [8], which in some West African foci have been reached after 14–17 years of annual (or biannual) ivermectin distribution [3,4]. This threshold was also exceeded in Asubende, which by the time of our study had received 24 years of ivermectin. Clear interpretation of this result is difficult since it is based on one infective fly caught in a Bellec trap, and flies using local breeding sites may originate from afar. However, there is also evidence from other studies that transmission in Asubende is continuing at a rate of >40 L3/person/month in some months (F.D.B. Veriegh, pers. comm.). Similarly, after 15 [64] and 17 [65] years of CDTI in Cameroon, or 20 years in the Central African Republic [66] have not resulted in interruption of transmission. Due to these and similar studies, there is a strong call for introducing more frequent (e.g. biannual) ivermectin treatments (or other strategies) if elimination is to be attained [67].

### Fly Infections Are due to *O*. *volvulus*

In regions in North Cameroon, approximately 70–90% of the filarial larvae in *S*. *damnosum* s.l. caught biting man were *O*. *ochengi* [68,69]. Given that cattle are present in some of our study villages (e.g. Agborlekame (~300 cows) and Asukawkaw Ferry (~500 cows), that *S*. *damnosum* s.l. flies feed on a range of blood hosts, and that 20% of the infective flies were caught using cattle-baited tents, we anticipated that we might have identified cattle-borne *Onchocerca* species such as *O*. *ochengi* but we only found *O*. *volvulus*. Over three quarters of the larvae had definitive *O*. *volvulus* identifications, and 96% of the unidentified larvae were from blackflies which had also contained known *O*. *volvulus* (of the same larval stage). No other species were identified and we are therefore confident that all of the *Onchocerca* larvae originated from flies infected with *O*. *volvulus*. This indicates active onchocerciasis transmission from humans to flies (early larval stages or infective flies attempting to feed on cattle) and from flies to humans (infective larvae in flies attempting to feed on humans). During the OCP, transmission potentials had been initially calculated on the assumption that all larvae would be *O*. *volvulus*; these ‘crude’ transmission potentials were subsequently corrected when tools for molecular identification of parasite larvae became available revealing that a geographically variable proportion of infective flies harboured non-*volvulus Onchocerca* spp. of zoonotic origin [70].

### Factors Impacting on Persistent Transmission

In 1980 (pre-ivermectin and pre-vector control), over 75% of the Asubende population were infected with microfilariae, and in 1987, prior to the ivermectin community trials, an infection prevalence of 80% was recorded [16], only slightly higher than that of Agborlekame (both in the Brong-Ahafo region). These communities were highly hyperendemic at baseline. The absence of infective flies observed at Agborlekame may be attributable to our low sample sizes, and/or recent treatment, rather than true lack of transmission. This conjecture is supported by on-going entomological studies (F.B.D. Veriegh pers. comm.) indicating high levels of L3 infections in flies from Agborlekame reaching 68 L3/person/month. This is further supported by our molecular analyses of fly abdomens, which revealed one infected fly in 83 flies analysed. At Asubende, biting rates have returned to pre-vector control levels [26], suggesting ecological conditions propitious for continuing transmission. Asubende has received regular annual treatment since 1987, and bi-annual treatment since 2009, with the most recent treatment round just 2 months before our sample collection. The village had a population of only 88 inhabitants at the time of sampling, and inspection of the community distributor’s notebooks and district records indicated a high therapeutic coverage. Therefore, in addition to the return of high biting rates and the possibility of infective flies migrating into the area [71], the potential for sub-optimal responses to ivermectin, perhaps suggesting decreased drug susceptibility, cannot be ignored. After 20 years of annual ivermectin administration, epidemiological assessments in 19 communities in Ghana, including Asubende, indicated a persistent reservoir of microfilarial infection [18,20].

In contrast, in the three Volta Region villages, transmission was low, despite a shorter history of vector control and ivermectin treatment than in Brong-Ahafo. The lack of infections may be attributable to the success of the OCP vector control strategy, which eliminated the Djodji form of *S*. *sanctipauli* [72], one of the *S*. *damnosum* complex species with the highest vector competence. Previous studies had shown that the Djodji form of *S*. *sanctipauli* carried, on average, three times as many L3 larvae per 1,000 biting flies as *S*. *squamosum* [73]. The reduction in biting rates associated with the disappearance of the Djodji form of *S*. *sanctipauli* [26] may also explain the reduction in transmission. Ivermectin treatment records also indicate that Pillar 83 had repeated ivermectin treatments in the years from 1993 to 1997 (potentially rapidly reducing levels of transmission in this community at the early stages of ivermectin control), followed by annual CDTI.

At Bosomase and Gyankobaa, which never received vector control and were incorporated into CDTI only recently, high levels of active transmission are still occurring, despite their lower baseline levels of infection intensity and prevalence, and current biannual or annual ivermectin treatment. In Gyankobaa, the most recent round of ivermectin distribution had taken place over a year before our sample collection date, providing ample opportunity for the reappearance of microfilariae in the hosts’ skin and their ensuing transmission [74,75]. In Bosomase, the high infection levels observed in the wet season in August 2009 are probably explicable by the missed annual treatment in that year, highlighting the importance of understanding the programmatic determinants of persistent transmission.

### The Role of Simuliid Species Composition and Vectorial Competence and Capacity

The transmission in the dry season of 2010 at Bosomase is of concern, with flies collected just one month after ivermectin treatment. However, seasonal variations (transmission levels in the 2009 wet season were higher than in the 2010 dry season), and in vector species composition and competence may also play a role in explaining the reported transmission patterns.

In the dry season, monthly transmission potentials were driven by *S*. *yahense*, with a higher number of L3s per infective fly than the extant form of *S*. *sanctipauli*. In contrast, the higher monthly infective biting rates in the wet season were driven by higher numbers of infective *S*. *sanctipauli* flies, despite their lower numbers of L3s per infective fly. Although not as anthropophagic and efficient a vector as the eliminated Djodji form, the forest form of *S*. *sanctipauli* has previously been demonstrated to be a highly efficient vector. In an area environmentally similar to, and just north of, Bosomase, a mean of 377 L3 in 1,000 parous flies, and 122 L3 per 1,000 biting flies (with 44% of parous flies infected) were recorded [76]. Even higher values, of 616 L3 per 1,000 parous flies have been reported in other African localities [56]. Overall, we observed lower infection rates than these, potentially due to the high therapeutic coverage of annual CDTI in this community. However, some reductions attributed to CDTI may actually be due to river pollution, lowering fly breeding success and associated transmission, particularly for *S*. *sanctipauli* [77], further supporting our previous biting rate findings and potential factors involved [26].

The influence of vector competence on transmission observed in Bosomase was also seen in Gyankobaa, where *S*. *yahense*, and to a lesser extent *S*. *squamosum*, were responsible for lower monthly transmission potentials due to lower biting rates and parous biting rates. In contrast, the forest form of *S*. *sanctipauli*, contributed to high numbers of L3/person/month due to high biting rates. Consequently, although both Bosomase and Gyankobaa have a shorter history of CDTI, the high transmission parameters recorded here for the vector species prevailing in this area must be emphasised. In Gyankobaa, infection levels (numbers of L3/1,000 parous flies) were 129 times as high, and in Bosomase, 291 to 365 times as high, as the WHO threshold. In both localities, the greatest proportion of L3 were found in *S*. *sanctipauli*, a species poorly or not at all represented in current transmission models.

Transmission models for African onchocerciasis have been mostly parameterised using *S*. *damnosum* s.s.*/S*. *sirbanum* data [6,63,78,79,80,81,82,83] to reflect transmission dynamics in savannah areas suffering from severe ocular sequelae due to onchocerciasis. Exceptions to these models are the studies by Davies (1993) [84], based on transmission of forest onchocerciasis by *S*. *soubrense* B *sensu Post*; some quantitative analyses on other *S*. *damnosum* complex species, including *S*. *leonense* and *S*. *squamosum* B [85,86], and the recent modelling study of the effect of climate change on onchocerciasis transmission in Ghana and Liberia, including *S*. *soubrense* [87]. Our findings highlight that data on vector competence and vectorial capacity for *O*. *volvulus* for other important vector species are crucially needed, particularly as regions with diverse and seasonally varying simuliid vector composition strive towards elimination.

### Implications of Host-Dependent and Host-Independent Trapping Methods for Transmission Monitoring and Surveillance

Approximately 40% of the flies were caught on Bellec traps, a similar proportion to that caught by the traditional OCP vector collector method, resulting in roughly equal numbers caught by host-independent and host-dependent methods. Light traps performed poorly, despite previous success at trapping *S*. *squamosum* [29] and other members of the *S*. *damnosum* complex [30] in Ghana. The prevalence of infected and infective flies, assessed by dissection, was similar among our host-dependent and host-independent catching techniques. Bellec-caught flies had higher infection prevalence, measured by DNA analyses of the abdomens, than the vector collector-caught flies. Positive abdomens in ovipositing flies could originate from microfilariae ingested with the blood meal (that did not escape the peritrophic matrix)—indicating transmission from humans to flies, and/or from L3 larvae migrating out of the thorax—indicating potential transmission from flies to humans. These results suggest that using oviposition (Bellec) traps in breeding sites along rivers close to villages, could augment (and perhaps replace) the more labour-intensive methods of human vector collection for monitoring vector infection levels. Large numbers of flies are required by techniques such as pool-screening [88], and with decreasing infection rates, the numbers to power transmission studies seeking to quantify reductions in transmission may need to be even larger [89]. Potential replacements for human landing catches, such as the Esperanza Window Trap, have been developed for *S*. *ochraceum* s.l. (the vector in Mexico and Guatemala) [90,91] and evaluated for host-seeking flies in Africa [92]. Oviposition traps have the added advantage that even nulliparous flies could contribute to the quantification of infection in thoraces, as sufficient time between an infected bite and oviposition elapses allowing any potential microfilariae to establish as L1s within the flies. The *O*. *volvulus* larvae thus collected could also be tested for ivermectin resistance markers once field probes are developed, helping in the monitoring and evaluation of transmission and of the potential spread of decreased ivermectin efficacy. This will become particularly pertinent with the increasing need for large-scale entomological evaluation of interventions as programmes strive for elimination, which will raise ethical concerns surrounding the widespread use of human landing catches. The host-independent Bellec traps could also be used in wider geographical perimeters during the post-MDA surveillance phase to complement more human exposure-focused methods in sentinel sites.

### Study Limitations

As vector competence is known to vary between seasons [93], blackfly collection was performed in both wet and dry seasons at five of the seven locations. (Due to incorporation at a later stage in the study of Asubende and Agborlekame, data were only collected during the dry season in these communities.) However, due to low blackfly catches at four of the study locations in one or the other of the seasons, Bosomase was the only location where substantial data were collected during both seasons. Although this reflects a lack of biting or ovipositing blackflies at the sampling times in these localities during these seasons, our results may not reflect true absence of simuliids and of any associated transmission for the whole season. This is particularly highlighted by our inability to detect *Onchocerca* larvae at Agborlekame, despite recent observations of on-going transmission (F.B.D. Veriegh, pers. comm.). Indeed, when blackfly abdomens were analysed, at least one positive result was obtained for *O*. *volvulus* infection in each of the villages assessed, indicating some level of active transmission. A potential limitation of analysing fly abdomens by molecular means is that higher levels of infection in vector collector-caught flies might be expected if any of the vector collectors caught the flies after the start of feeding and were themselves infected with microfilariae. There was no evidence that *O*. *volvulus*-positive abdomens were caused by microfilariae from the vector collectors as proportions of infected blackfly abdomens were significantly lower in the vector collector-caught flies than in those obtained by the remaining trapping methods.

### Summary

1. Evidence of active *O*. *volvulus* transmission has been documented in seven Ghanaian communities with different histories of vector and ivermectin control.
2. Levels of *O*. *volvulus* infection with L3 larvae in parous flies were well above the WHO threshold for transmission control in two of these communities (Bosomase and Gyankobaa).
3. At Asubende, an infective ovipositing fly was found. In this locality, high vector density was recorded, with biting rates similar to pre vector control levels. Ecological conditions propitious to intense transmission remain in this formerly highly hyperendemic focus.
4. Complementary or alternative treatment strategies may be required to interrupt transmission in these areas, particularly as the WHO roadmap aims for elimination of the infection reservoir in certain African countries by 2020.
5. The local and seasonal mix of vector species influence transmission indices. Transmission dynamics models should be parameterised with vectorial competence and capacity data according to the local *S*. *damnosum* s.l. species composition. Each species will contribute differentially to overall transmission potentials and may respond differently to control interventions.
6. The use of oviposition (Bellec) traps could be used to enhance (or ultimately replace) the traditional OCP vector collection methods for the purposes of transmission monitoring and evaluation, parasite genetic studies for ivermectin susceptibility or determination of the extent of transmission zones, and post-MDA surveillance.

## Supporting Information

S1 Table Monthly biting rates by locality, season, trapping technique, host and species. (DOCX)

S2 Table Summary of all dissections for *Onchocerca* spp. larvae. (DOCX)
