# Supplementary material for: Onchocerciasis Transmission in Ghana: Persistence under Different Control Strategies and the Role of the Simuliid Vectors
Source: PLoS Negl Trop Dis. 2015 Apr 21;9(4):e0003688. doi: 10.1371/journal.pntd.0003688 (PMC4405193; doi:10.1371/journal.pntd.0003688)
Supplement: S1 Table — (DOCX) [file pntd.0003688.s001.docx]

Table S1: Monthly biting rates (MBRs) by locality, season, trapping technique, host and species.

| **Region** | **Village** | **Season** | **Trapping Method** | ***S. damnosum* s.l.** | ***S.  damnosum* s.s. */ S. sirbanum*** | ***S. soubrense* Beffa form** | ***S. squamosum*** | ***S. yahense*** | ***S. sanctipauli*** |
| --- | --- | --- | --- | --- | --- | --- | --- | --- | --- |
| **Brong-Ahafo** | **Asubende** | **Dry 2011** | **V/C** | 2,061 | 2,061 | - | - | - | - |
|  |  |  | **Human-tent** | 198 | 198 | - | - | - | - |
|  |  |  | **Cow-tent** | 238 | 238 | - | - | - | - |
|  | **Agborlekame** | **Dry 2010** | **V/C** | 775 | 775 | - | - | - | - |
|  |  |  | **Human-tent** | 32 | 32 | - | - | - | - |
|  |  |  | **Cow-tent** | 0 | 0 | - | - | - | - |
| **Volta** | **Asukawkaw Ferry** | **Wet 2009** | **V/C** | - | - | - | - | - | - |
|  |  |  | **Human-tent** | 1,349 | 0 | 1,241 | 108 | - | - |
|  |  |  | **Cow-tent** | 208 | 0 | 185 | 23 | - | - |
|  |  | **Dry 2010** | **V/C** | 5,777 | 1,955 | 320 | 3,502 | - | - |
|  |  |  | **Human-tent** | 1,057 | 122 | 137 | 798 | - | - |
|  |  |  | **Cow-tent** | 951 | 38 | 198 | 715 | - | - |
|  |  | **Dry 2011** | **V/C** | 5,429 | 1,545 | 247 | 3,637 | - | - |
|  |  |  | **Human-tent** | 760 | 131 | 25 | 605 | - | - |
|  |  |  | **Cow-tent** | 373 | 81 | 32 | 260 | - | - |
|  | **Dodi Papase** | **Wet 2009** | **V/C** | - | - | - | - | - | - |
|  |  |  | **Human-tent** | 519 | 7 | 0 | 512 | - | - |
|  |  |  | **Cow-tent** | 321 | 7 | 7 | 307 | - | - |
|  |  | **Dry 2010** | **V/C** | 2,357 | 137 | 83 | 2,136 | - | - |
|  |  |  | **Human-tent** | 312 | 0 | 15 | 297 | - | - |
|  |  |  | **Cow-tent** | 117 | 0 | 0 | 117 | - | - |
|  |  | **Dry 2011** | **V/C** | 4,371 | 367 | 22 | 3,971 | - | - |
|  |  |  | **Human-tent** | 1,079 | 60 | 12 | 1,007 | - | - |
|  |  |  | **Cow-tent** | 378 | 43 | 0 | 324 | 11 | - |
|  | **Pillar 83/Djodji** | **Wet 2009** | **V/C** | - | - | - | - | - | - |
|  |  |  | **Human-tent** | 15 | - | - | 15 | - | - |
|  |  |  | **Cow-tent** | 91 | - | - | 91 | - | - |
|  |  | **Dry 2010** | **V/C** | 7,171 | 321 | - | 6,776 | - | - |
|  |  |  | **Human-tent** | 494 | 0 | 0 | 494 | - | - |
|  |  |  | **Cow-tent** | 586 | 0 | 8 | 578 | - | - |
|  |  | **Dry 2011** | **V/C** | 9,329 | 96 | 20 | 9,167 | 20 | - |
|  |  |  | **Human-tent** | 2,748 | 159 | 0 | 2,589 | - | - |
|  |  |  | **Cow-tent** | 1,298 | 152 | 0 | 1,146 | - | - |
| **Western** | **Bosomase^a^** | **Wet 2009** | **V/C** | 5,481 | - | - | - | - | 5481 |
|  |  |  | **Human-tent** | 1,447 | - | - | 7 | - | 1440 |
|  |  |  | **Cow-tent** | 1,613 | - | - | - | - | 1613 |
|  |  | **Dry 2010** | **V/C** | 1,209 | - | - | - | 385 | 824 |
|  |  |  | **Human-tent** | 1,011 | - | - | - | 631 | 380 |
|  |  |  | **Cow-tent** | 731 | - | - | - | 544 | 187 |
| **Ashanti** | **Gyankobaa^b^** | **Wet 2009** | **V/C** | 4,121 | 247 | - | 486 | 0 | 3388 |
|  |  |  | **Human-tent** | 4,910 | 33 | - | 9 | 108 | 4760 |
|  |  |  | **Cow-tent** | 4,366 | 120 | - | 0 | 46 | 4200 |

Calculated monthly biting rates (MBR) of host-seeking blackflies by locality, season, trapping technique and species. The values for vector collector (V/C) and Human-tent indicate number of bites per person per month; the values for Cow-tent are bites per cow per month; the values for Host-seeking are the average values per host per month. **^a^**Bosomase in the Dry season in 2011 for 11hrs, V/C = 2 *S. sanctipauli* = 61bites/person/month. **^b^**Gyankobaa in the Dry season in 2010 for 7hrs, Cow-Tent = 2 *S. damnosum* s.s.*/S. sirbanum* = 96 flies/cow/month.
