# Supplementary material for: Onchocerciasis Transmission in Ghana: Persistence under Different Control Strategies and the Role of the Simuliid Vectors
Source: PLoS Negl Trop Dis. 2015 Apr 21;9(4):e0003688. doi: 10.1371/journal.pntd.0003688 (PMC4405193; doi:10.1371/journal.pntd.0003688)
Supplement: S2 Table — (DOCX) [file pntd.0003688.s002.docx]

Table S2: Summary of all dissections for *Onchocerca* spp. larvae.

| **Region** | **Village** | **Season** | **Trapping Method** | **No. of flies dissected** | **No. of flies with L1, L2 or L3s** | **No. of flies with L3s in head** | **No. of flies with L3s in head and/or thorax** | **Total No. of L3s (head only)** | **Total No. of L3s (head and thorax)** | **Proportion (%) of flies infected (all larval stages)**  **(95%CI)** | **Proportion (%) of flies infective (L3s in head)**  **(95%CI)** | **Proportion (%) of flies infective**  **(L3s in head and thorax) (95%CI)** |
| --- | --- | --- | --- | --- | --- | --- | --- | --- | --- | --- | --- | --- |
| **Brong-Ahafo** | **Asubende** | **Dry**  **Feb 2011** | **V/C** | 362 | 0 | 0 | 0 | 0 | 0 | 0 (0-1.0) | 0 (0-1.0) | 0 (0-1.0) |
|  |  |  | **Human-tent** | 34 | 0 | 0 | 0 | 0 | 0 | 0 (0-10.3) | 0 (0-10.3) | 0 (0-10.3) |
|  |  |  | **Cow-tent** | 42 | 0 | 0 | 0 | 0 | 0 | 0 (0-8.4) | 0 (0-8.4) | 0 (0-8.4) |
|  |  |  | **Bellec traps** | 741 | 1 | 0 | 1 | 0 | 1 | 0.1 (0.0-0.8) | 0 (0-0.5) | 0.1 (0.0-0.8) |
|  |  |  | **Light traps** | 0 | NA | NA | NA | NA | NA | NA | NA | NA |
|  |  |  | **Total** | **1179** | **1** | **0** | **1** | **0** | **1** | **0.1 (0.0-0.5)** | **0 (0-0.3)** | **0.1 (0.0-0.5)** |
|  | **Agborlekame** | **Dry**  **Feb 2010** | **V/C** | 73 | 0 | 0 | 0 | 0 | 0 | 0 (0-4.9) | 0 (0-4.9) | 0 (0-4.9) |
|  |  |  | **Human-tent** | 6 | 0 | 0 | 0 | 0 | 0 | 0 (0-45.9) | 0 (0-45.9) | 0 (0-45.9) |
|  |  |  | **Cow-tent** | 0 | NA | NA | NA | NA | NA | NA | NA | NA |
|  |  |  | **Bellec traps** | 83 | 0 | 0 | 0 | 0 | 0 | 0 (0-4.4) | 0 (0-4.4) | 0 (0-4.4) |
|  |  |  | **Light traps** | 0 | NA | NA | NA | NA | NA | NA | NA | NA |
|  |  |  | **Total** | **156** | **0** | **0** | **0** | **0** | **0** | **0 (0-2.3)** | **0 (0-2.3)** | **0 (0-2.3)** |
| **Volta** | **Asukawkaw Ferry** | **Wet**  **Aug 2009** | **V/C** | NA | NA | NA | NA | NA | NA | NA | NA | NA |
|  |  |  | **Human-tent** | 252 | 0 | 0 | 0 | 0 | 0 | 0 (0-1.5) | 0 (0-1.5) | 0 (0-1.5) |
|  |  |  | **Cow-tent** | 36 | 0 | 0 | 0 | 0 | 0 | 0 (0-9.7) | 0 (0-9.7) | 0 (0-9.7) |
|  |  |  | **Bellec traps** | 80 | 0 | 0 | 0 | 0 | 0 | 0 (0-4.5) | 0 (0-4.5) | 0 (0-4.5) |
|  |  |  | **Light traps** | 2 | 0 | 0 | 0 | 0 | 0 | 0 (0-84.2) | 0 (0-84.2) | 0 (0-84.2) |
|  |  |  | **Total** | **370** | **0** | **0** | **0** | **0** | **0** | **0 (0-1.0)** | **0 (0-1.0)** | **0 (0-1.0)** |
|  |  | **Dry**  **March 2010** | **V/C** | 694 | 0 | 0 | 0 | 0 | 0 | 0 (0-0.5) | 0 (0-0.5) | 0 (0-0.5) |
|  |  |  | **Human-tent** | 125 | 0 | 0 | 0 | 0 | 0 | 0 (0-2.9) | 0 (0-2.9) | 0 (0-2.9) |
|  |  |  | **Cow-tent** | 118 | 1 | 0 | 0 | 0 | 0 | 0.8 (0-4.6) | 0 (0-3.1) | 0 (0-3.1) |
|  |  |  | **Bellec traps** | 93 | 0 | 0 | 0 | 0 | 0 | 0 (0-3.9) | 0 (0-3.9) | 0 (0-3.9) |
|  |  |  | **Light traps** | 0 | NA | NA | NA | NA | NA | NA | NA | NA |
|  |  |  | **Total** | **1030** | **1** | **0** | **0** | **0** | **0** | **0.1 (0.0-0.5)** | **0 (0-0.4)** | **0 (0-0.4)** |
|  |  | **Dry**  **Feb 2011** | **V/C** | 641 | 0 | 0 | 0 | 0 | 0 | 0 (0-0.6) | 0 (0-0.6) | 0 (0-0.6) |
|  |  |  | **Human-tent** | 93 | 0 | 0 | 0 | 0 | 0 | 0 (0-3.9) | 0 (0-3.9) | 0 (0-3.9) |
|  |  |  | **Cow-tent** | 47 | 0 | 0 | 0 | 0 | 0 | 0 (0-7.6) | 0 (0-7.6) | 0 (0-7.6) |
|  |  |  | **Bellec traps** | 61 | 0 | 0 | 0 | 0 | 0 | 0 (0-5.9) | 0 (0-5.9) | 0 (0-5.9) |
|  |  |  | **Light traps** | 0 | NA | NA | NA | NA | NA | NA | NA | NA |
|  |  |  | **Total** | **842** | **0** | **0** | **0** | **0** | **0** | **0 (0-0.4)** | **0 (0-0.4)** | **0 (0-0.4)** |
|  | **Dodi Papase** | **Wet**  **Aug 2009** | **V/C** | 86 | 0 | 0 | 0 | 0 | 0 | 0 (0-4.2) | 0 (0-4.2) | 0 (0-4.2) |
|  |  |  | **Human-tent** | 77 | 0 | 0 | 0 | 0 | 0 | 0 (0-4.7) | 0 (0-4.7) | 0 (0-4.7) |
|  |  |  | **Cow-tent** | 46 | 0 | 0 | 0 | 0 | 0 | 0 (0-7.7) | 0 (0-7.7) | 0 (0-7.7) |
|  |  |  | **Bellec traps** | 15 | 0 | 0 | 0 | 0 | 0 | 0 (0-21.8) | 0 (0-21.8) | 0 (0-21.8) |
|  |  |  | **Light traps** | 0 | NA | NA | NA | NA | NA | NA | NA | NA |
|  |  |  | **Total** | **225** | **0** | **0** | **0** | **0** | **0** | **0 (0-1.6)** | **0 (0-1.6)** | **0 (0-1.6)** |
|  |  | **Dry**  **March 2010** | **V/C** | 309 | 0 | 0 | 0 | 0 | 0 | 0 (0-1.2) | 0 (0-1.2) | 0 (0-1.2) |
|  |  |  | **Human-tent** | 41 | 0 | 0 | 0 | 0 | 0 | 0 (0-8.6) | 0 (0-8.6) | 0 (0-8.6) |
|  |  |  | **Cow-tent** | 13 | 0 | 0 | 0 | 0 | 0 | 0 (0-24.7) | 0 (0-24.7) | 0 (0-24.7) |
|  |  |  | **Bellec traps** | 25 | 0 | 0 | 0 | 0 | 0 | 0 (0-13.7) | 0 (0-13.7) | 0 (0-13.7) |
|  |  |  | **Light traps** | 0 | NA | NA | NA | NA | NA | NA | NA | NA |
|  |  |  | **Total** | **388** | **0** | **0** | **0** | **0** | **0** | **0 (0-1.0)** | **0 (0-1.0)** | **0 (0-1.0)** |
|  |  | **Dry**  **Feb 2011** | **V/C** | 394 | 0 | 0 | 0 | 0 | 0 | 0 (0-0.9) | 0 (0-0.9) | 0 (0-0.9) |
|  |  |  | **Human-tent** | 86 | 0 | 0 | 0 | 0 | 0 | 0 (0-4.2) | 0 (0-4.2) | 0 (0-4.2) |
|  |  |  | **Cow-tent** | 36 | 0 | 0 | 0 | 0 | 0 | 0 (0-9.7) | 0 (0-9.7) | 0 (0-9.7) |
|  |  |  | **Bellec traps** | 66 | 0 | 0 | 0 | 0 | 0 | 0 (0-5.4) | 0 (0-5.4) | 0 (0-5.4) |
|  |  |  | **Light traps** | 0 | NA | NA | NA | NA | NA | NA | NA | NA |
|  |  |  | **Total** | **582** | **0** | **0** | **0** | **0** | **0** | **0 (0-0.6)** | **0 (0-0.6)** | **0 (0-0.6)** |
|  | **Pillar 83/Djodji** | **Wet**  **July 2009** | **V/C** | NA | NA | NA | NA | NA | NA | NA | NA | NA |
|  |  |  | **Human-tent** | 1 | 0 | 0 | 0 | 0 | 0 | 0 (0-97.5) | 0 (0-97.5) | 0 (0-97.5) |
|  |  |  | **Cow-tent** | 9 | 0 | 0 | 0 | 0 | 0 | 0 (0-33.6) | 0 (0-33.6) | 0 (0-33.6) |
|  |  |  | **Bellec traps** | 8 | 0 | 0 | 0 | 0 | 0 | 0 (0-36.9) | 0 (0-36.9) | 0 (0-36.9) |
|  |  |  | **Light traps** | 29 | 0 | 0 | 0 | 0 | 0 | 0 (0-11.9) | 0 (0-11.9) | 0 (0-11.9) |
|  |  |  | **Total** | **47** | **0** | **0** | **0** | **0** | **0** | **0 (0-7.6)** | **0 (0-7.6)** | **0 (0-7.6)** |
|  |  | **Dry**  **March 2010** | **V/C** | 577 | 0 | 0 | 0 | 0 | 0 | 0 (0-0.6) | 0 (0-0.6) | 0 (0-0.6) |
|  |  |  | **Human-tent** | 62 | 0 | 0 | 0 | 0 | 0 | 0 (0-5.8) | 0 (0-5.8) | 0 (0-5.8) |
|  |  |  | **Cow-tent** | 75 | 0 | 0 | 0 | 0 | 0 | 0 (0-4.8) | 0 (0-4.8) | 0 (0-4.8) |
|  |  |  | **Bellec traps** | 1268 | 0 | 0 | 0 | 0 | 0 | 0 (0-0.3) | 0 (0-0.3) | 0 (0-0.3) |
|  |  |  | **Light traps** | 22 | 0 | 0 | 0 | 0 | 0 | 0 (0-15.4) | 0 (0-15.4) | 0 (0-15.4) |
|  |  |  | **Total** | **2004** | **0** | **0** | **0** | **0** | **0** | **0 (0-0.2)** | **0 (0-0.2)** | **0 (0-0.2)** |
|  |  | **Dry**  **Feb 2011** | **V/C** | 829 | 0 | 0 | 0 | 0 | 0 | 0 (0-0.4) | 0 (0-0.4) | 0 (0-0.4) |
|  |  |  | **Human-tent** | 273 | 0 | 0 | 0 | 0 | 0 | 0 (0-1.3) | 0 (0-1.3) | 0 (0-1.3) |
|  |  |  | **Cow-tent** | 965 | 0 | 0 | 0 | 0 | 0 | 0 (0-0.4) | 0 (0-0.4) | 0 (0-0.4) |
|  |  |  | **Bellec traps** | 476 | 0 | 0 | 0 | 0 | 0 | 0 (0-0.8) | 0 (0-0.8) | 0 (0-0.8) |
|  |  |  | **Light traps** | 9 | 0 | 0 | 0 | 0 | 0 | 0 (0-33.6) | 0 (0-33.6) | 0 (0-33.6) |
|  |  |  | **Total** | **2552** | 0 | 0 | 0 | 0 | 0 | **0 (0-0.1)** | **0 (0-0.1)** | **0 (0-0.1)** |
| **Western** | **Bosomase** | **Dry**  **Feb 2006** | **Bellec** | 3352 | 23 | 20 | 20 | 23 | 23 | 0.7 (0-1.0) | 0.7 (0-1.0) | 0.7 (0-1.0) |
|  |  |  | **Total** | **3352** | **23** | **20** | **20** | **23** | **23** | **0.7 (0-1.0)** | **0.7 (0-1.0)** | **0.7 (0-1.0)** |
|  |  | **Wet**  **Aug 2009** | **V/C** | 397 | 6 | 2 | 3 | 8 | 30 | 5.8 (1.0-8.6) | 0.5 (0.1-1.8) | 0.8 (0.1-2.2) |
|  |  |  | **Human-tent** | 188 | 0 | 0 | 0 | 0 | 0 | 0 (0-1.9) | 0 (0-1.9) | 0 (0-1.9) |
|  |  |  | **Cow-tent** | 191 | 5 | 1 | 2 | 4 | 7 | 2.6 (0.4-6.0) | 0.5 (0.1-2.9) | 1.0 (0.2-3.7) |
|  |  |  | **Bellec traps** | 54 | 1 | 1 | 1 | 1 | 1 | 1.9 (0.3-9.9) | 1.9 (0.3-9.9) | 1.9 (0.3-9.9) |
|  |  |  | **Light traps** | 7 | 0 | 0 | 0 | 0 | 0 | 0 (0-41.0) | 0 (0-41.0) | 0 (0-41.0) |
|  |  |  | **Total** | **837** | **12** | **4** | **6** | **13** | **38** | **1.4 (0.2-2.5)** | **0.5 (0.1-1.2)** | **0.7 (0.1-1.6)** |
|  |  | **Dry**  **Feb 2010** | **V/C** | 152 | 3 | 0 | 0 | 0 | 0 | 2.0 (0.3-5.7) | 0 (0-2.4) | 0 (0-2.4) |
|  |  |  | **Human-tent** | 145 | 4 | 0 | 1 | 0 | 1 | 2.8 (0.5-6.9) | 0 (0-2.5) | 0.7 (0.1-3.8) |
|  |  |  | **Cow-tent** | 73 | 3 | 2 | 3 | 26 | 37 | 4.1 (0.7-11.5) | 2.7 (0.5-9.6) | 4.1 (0.7-11.5) |
|  |  |  | **Bellec traps** | 355 | 4 | 4 | 4 | 4 | 6 | 1.1 (0.2-2.8) | 1.1 (0.2-2.8) | 1.1 (0.2-2.8) |
|  |  |  | **Light traps** | 0 | NA | NA | NA | NA | NA | NA | NA | NA |
|  |  |  | **Total** | **725** | **14** | **6** | **8** | **30** | **44** | **1.9 (0.3-3.2)** | **0.8 (0.1-1.8)** | **1.1 (0.2-2.1)** |
| **Ashanti** | **Gyankobaa** | **Wet**  **Aug 2009** | **V/C** | 540 | 16 | 4 | 5 | 8 | 11 | 3.0 (0.5-4.8) | 0.7 (0.1-1.9) | 0.9 (0.3-2.1) |
|  |  |  | **Human-tent** | 571 | 10 | 2 | 5 | 16 | 27 | 1.8 (0.3-3.2) | 0.4 (0.0-1.3) | 0.9 (0.2-2.0) |
|  |  |  | **Cow-tent** | 441 | 11 | 5 | 5 | 12 | 14 | 2.5 (0.4-4.4) | 1.1 (0.2-2.6) | 1.1 (0.2-2.6) |
|  |  |  | **Bellec traps** | 524 | 6 | 2 | 5 | 4 | 14 | 1.1 (0.2-2.5) | 0.4 (0.1-1.4) | 1.0 (0.2-2.2) |
|  |  |  | **Light traps** | 103 | 3 | 2 | 3 | 28 | 40 | 2.9 (0.5-8.3) | 1.9 (0.3-6.8) | 2.9 (0.5-8.3) |
|  |  |  | **Total** | **2179** | **46** | **15** | **24** | **68** | **106** | **2.1 (0.4-2.8)** | **0.7 (0.1-1.1)** | **1.1 (0.2-1.6)** |
|  |  | **Dry**  **Feb 2010** | **V/C** | 0 | NA | NA | NA | NA | NA | NA | NA | NA |
|  |  |  | **Human-tent** | 0 | NA | NA | NA | NA | NA | NA | NA | NA |
|  |  |  | **Cow-tent** | 1 | 0 | 0 | 0 | 0 | 0 | 0 (0-97.5) | 0 (0-97.5) | 0 (0-97.5) |
|  |  |  | **Bellec traps** | 10 | 0 | 0 | 0 | 0 | 0 | 0 (0-30.9) | 0 (0-30.9) | 0 (0-30.9) |
|  |  |  | **Light traps** | 0 | NA | NA | NA | NA | NA | NA | NA | NA |
|  |  |  | **Total** | **11** | **0** | **0** | **0** | **0** | **0** | **0 (0-28.5)** | **0 (0-28.5)** | **0 (0-28.5)** |
|  |  | **Dry**  **Feb 2011** | **V/C** | 0 | NA | NA | NA | NA | NA | NA | NA | NA |
|  |  |  | **Human-tent** | 0 | NA | NA | NA | NA | NA | NA | NA | NA |
|  |  |  | **Cow-tent** | 0 | NA | NA | NA | NA | NA | NA | NA | NA |
|  |  |  | **Bellec traps** | 1 | 0 | 0 | 0 | 0 | 0 | 0 (0-97.5) | 0 (0-97.5) | 0 (0-97.5) |
|  |  |  | **Light traps** | 0 | NA | NA | NA | NA | NA | NA | NA | NA |
|  |  |  | **Total** | **1** | **0** | 0 | 0 | 0 | 0 | 0 (0-97.5) | 0 (0-97.5) | 0 (0-97.5) |
